# Comparative genome‐wide analysis reveals that *Burkholderia contaminans* MS14 possesses multiple antimicrobial biosynthesis genes but not major genetic loci required for pathogenesis

**DOI:** 10.1002/mbo3.333

**Published:** 2016-01-14

**Authors:** Peng Deng, Xiaoqiang Wang, Sonya M. Baird, Kurt C. Showmaker, Leif Smith, Daniel G. Peterson, Shien Lu

**Affiliations:** ^1^ Departments of Biochemistry, Molecular BiologyEntomology and Plant PathologyMississippi State UniversityMississippi stateMississippi; ^2^ Institute for GenomicsBiocomputing and BiotechnologyMississippi State UniversityMississippi stateMississippi; ^3^ Department of BiologyTexas A&M UniversityCollege StationTexas

**Keywords:** Antimicrobial, *Burkholderia contaminans *MS14, comparative genomics, virulence, whole genome sequencing.

## Abstract

*Burkholderia contaminans* MS14 shows significant antimicrobial activities against plant and animal pathogenic fungi and bacteria. The antifungal agent occidiofungin produced by MS14 has great potential for development of biopesticides and pharmaceutical drugs. However, the use of *Burkholderia* species as biocontrol agent in agriculture is restricted due to the difficulties in distinguishing between plant growth‐promoting bacteria and the pathogenic bacteria. The complete MS14 genome was sequenced and analyzed to find what beneficial and virulence‐related genes it harbors. The phylogenetic relatedness of *B. contaminans* MS14 and other 17 *Burkholderia* species was also analyzed. To research MS14′s potential virulence, the gene regions related to the antibiotic production, antibiotic resistance, and virulence were compared between MS14 and other *Burkholderia* genomes. The genome of *B. contaminans* MS14 was sequenced and annotated. The genomic analyses reveal the presence of multiple gene sets for antimicrobial biosynthesis, which contribute to its antimicrobial activities. BLAST results indicate that the MS14 genome harbors a large number of unique regions. MS14 is closely related to another plant growth‐promoting *Burkholderia* strain *B. lata* 383 according to the average nucleotide identity data. Moreover, according to the phylogenetic analysis, plant growth‐promoting species isolated from soils and mammalian pathogenic species are clustered together, respectively. MS14 has multiple antimicrobial activity‐related genes identified from the genome, but it lacks key virulence‐related gene loci found in the pathogenic strains. Additionally, plant growth‐promoting *Burkholderia* species have one or more antimicrobial biosynthesis genes in their genomes as compared with nonplant growth‐promoting soil‐isolated *Burkholderia* species. On the other hand, pathogenic species harbor multiple virulence‐associated gene loci that are not present in nonpathogenic *Burkholderia* species. The MS14 genome as well as *Burkholderia* species genome show considerable diversity. Multiple antimicrobial agent biosynthesis genes were identified in the genome of plant growth‐promoting species of Burkholderia. In addition, by comparing to nonpathogenic *Burkholderia* species, pathogenic *Burkholderia* species have more characterized homologs of the gene loci known to contribute to pathogenicity and virulence to plant and animals.

## Background

*Burkholderia* is a gram‐negative, rod‐shaped, motile, and nonspore‐forming bacterium that has been identified in many diverse ecological niches (Francis et al. 2013). Currently, 88 species have been recognized in the genus *Burkholderia* (De Meyer et al. 2013). The ecological versatility of these bacteria is likely due to their unusually large genomes, which are often comprised of one or multiple large replicons with plasmids (Lessie et al. 1996). The bacterium has the ability to use a large array of carbon sources to synthesize secondary metabolites (el ‐Banna and Winkelmann 1998; Parke and Gurian‐Sherman 2001).

Most *Burkholderia* species isolated from soil are associated with plants. Some species are related to the promotion of plant growth and are considered to be plant growth‐promoting bacteria (PGPB). For example, *B. phenoliruptrix* and *B. phymatum* are effective in nitrogen fixing (Elliott et al. 2007; Zuleta et al. 2014), while *B. phytofirmans* induces larger root systems (Sessitsch et al. 2005). Interests in the use of *Burkholderia* species or their secondary metabolites in agriculture have increased. For example, the use of *Burkholderia cepacia* AMMDR1 could yield as efficient control of “damping‐off” disease caused by *Pythium* species and *Rhizoctonia solani* as that of the fungicide Captan (Heungens and Parke 2000; Parke and Gurian‐Sherman 2001). The relatively large *Burkholderia* genome harbors a large variety of antimicrobial biosynthesis genes. Occidiofungin (Gu et al. 2011; Ellis et al. 2012) produced by *B. contaminans* MS14 has significant antifungal activity. Pyrrolnitrin (el ‐Banna and Winkelmann 1998) was first identified as an antifungal antibiotic produced by *Pseudomonas* species and later found to be synthesized by several *Burkholderia cepacia* species. Pyoluteorin (Birchall et al. 1970) and lipopeptide AFC‐BC11 (Kang et al. 1998) are other antibiotics produced by *Burkholderia*. Siderophores are bacteriostatic agents that can inhibit pathogenic microorganism's growth by depleting iron in the soil (Hider and Kong 2010). Pyochelin (Serino et al. 1997) and ornibactin (Meyer et al. 1995) are common siderophores produced by *Burkholderia* species. On the other hand, plant‐pathogenic species of *Burkholderia* have also been identified, such as *B. gladioli* and *B. glumae*, which infect rice and other horticultural plants (Stoyanova et al. 2007; Ham et al. 2011). The commonly produced plant‐toxic secondary metabolites by *Burkholderia* species include polysaccharides and other toxins, such as rice grain rot and wilt causal agent toxoflavin (Latuasan and Berends 1961) and exopolysaccharide toxin cepacian (Ferreira et al. 2010) that contribute to the overall pathogenicity and success of the bacterium as a plant pathogen.

The potent antifungal occidiofungin was first identified from *B. contaminans* MS14 (Gu et al. 2011). *Burkholderia contaminans* MS14 was isolated from soil in Mississippi, it has a broad range of antifungal activities to plant and human pathogens by producing an oligopeptide occidiofungin (Lu et al. 2009). It is a novel fungicide that can significantly inhibit the growth of pathogens by interfering with cell wall synthesis or triggering apoptosis (Ellis et al. 2012; Emrick et al. 2013; Ravichandran et al. 2013). Genetically, the whole length of the *ocf* gene cluster required for production of occidiofungin has been characterized, which is composed of 16 ORFs (Gu et al. 2009). Among the 16 members of this cluster, *ocfD, ocfE, ocfF, ocfH*, and *ocfJ* were predicted to encode nonribosomal peptide synthesis (NRPS) or NRPS‐polyketide synthase (PKS), which are directly related to the biosynthesis of the antifungal compound occidiofungin. The genes *ocfA*,* ocfC, ocfK, ocfL, ocfM,* and *ocfN* were predicted to be involved in the secretion and modification of occidiofungin.

*Burkholderia cepacia* complex (Bcc) is a group of *Burkholderia* species that some are opportunistic bacteria and could cause lung disease in immunocompromised individuals (Mahenthiralingam et al. 2005). The Bcc group composed of nine different genomovars and at least 18 different species. These species include *B. cepacia*,* B. cenocepacia, B. multivorans*,* B. vietnamiensis*,* B. stabilis*,* B. ambifaria*,* B. dolosa*,* B. anthina*, and *B. pyrrocinia* (Lipuma 2005). *B. cepacia* is a common environmental species, but is also an important human pathogen which can create respiratory complications for cystic fibrosis (CF) patients (Mahenthiralingam et al. 2005). *B. cenocepacia* is a major CF pathogen and is responsible for 70% of the cases of Bcc infection (Mahenthiralingam et al. 2005). *B. multivorans* is the second most common Bcc species in CF infection (Mahenthiralingam et al. 2002), and some species like *B. dolosa* strains are frequently isolated from the CF patents (Vermis et al. 2004). Bcc infections contribute to the overall poor health of CF patients (Mahenthiralingam et al. 2002). It is important to be able to distinguish virulent Bcc species, as well as some other reported virulent *Burkholderia* species such as *B. pseudomallei*, from the less‐virulent soil‐isolated *Burkholderia* species.

Resistance to multiple antibiotics and disinfectants is very common among *Burkholderia* species. This antibiotic resistance feature makes them hard to treat and is crucial for human pathogenicity of Bcc species. There are several mechanisms that contribute to antibiotic resistance of the Bcc strains. First, efflux pumps are responsible for exclusion of antibiotics from the cell. Secondly, some *Burkholderia* species could significantly lower antimicrobial susceptibility by forming biofilm (Sawasdidoln et al. 2010) or by entering a nonreplicating state (Hamad et al. 2011). In addition, enzymatic inactivation of antibiotics either by modification or cleavage is a common resistance mechanism found in *Burkholderia* species (Tribuddharat et al. 2003).

Several well‐documented bacterial virulence features for mammalian pathogenesis are found in *Burkholderia* species. Cable pili and the 22‐kilodalton (KDa) adhesin are virulence factors associated with cepacia syndrome. These are required for *B. cenocepacia* to bind and cross the squamous epithelium leading to an intensified chronic infection (Urban et al. 2005). The periplasmic located superoxide dismutase (SOD) protects bacteria from oxidation by exogenously generated superoxide or peroxide (Keith and Valvano 2007). A 31.7 kb *B. cepacia* virulence genomic island (GIs) has been identified to harbor *B. cepacia* epidemic strain marker (BCESM) and possesses both virulence and metabolism‐associated genes (Baldwin et al. 2004). VgrG‐5 is a *Burkholderia* type VI secretion system 5‐associated protein, which is required for the full virulence of type VI secretion system 5 to induce multinucleate giant cell formation and full mammalian virulence (Schwarz et al. 2014).

In this study, we analyzed the phylogenetic relatedness of MS14 to 17 other *Burkholderia* species including plant pathogen, cystic fibrosis opportunistic, plant growth‐promoting strains, and other soil isolates. Being able to distinguish between safe environmental isolates of *Burkholderia* and a human pathogen is a current problem (Mahenthiralingam et al. 2008). Our analyses provide a whole‐genome approach to identify less‐virulent environmental isolates by comparing antibiotic biosynthesis, antibiotic resistance, and virulence loci among *Burkholderia* species. The results provide important information for evaluating *Burkholderia* species virulence according to their secondary metabolites production and virulence markers.

## Materials and Methods

### Genome sequencing of *B. contaminans* MS14

*B. contaminans* MS14 was cultured in nutrient broth yeast (NBY) extract medium (Vidaver 1967) overnight in a shaker at 28°C. The genomic DNA was extracted using the Wizard Genomic DNA Purification Kit (Promega Corporation, Madison, WI, USA) and was used for library construction with Illumina Genomic DNA Sample Preparation Kit (Illumina, CA, USA). Twelve standard libraries (with an average insert size of 400~700 bp) and one mate pair library (with an average insert size of 7000 bp) were prepared and sequenced on the Illumina MiSeq and HiSeq instrument according to the manufacturer's instructions. The genome was de novo assembled similar as described by Tim Durfee, etc. (Durfee et al. 2008) using DNAStar Seqman NGen (Version 12, DNASTAR, Inc. Madison, WI U.S.). Briefly, a standard 400 bp insertion library and the mate pair library were selected as the input data for the assembling. Forty million short reads were scanned and extracted from the raw data files as input data. The short reads were preprocessed by Seqman NGen to trim adaptors and filter low‐quality reads. Automatic Mer size was chosen and a minimum match percentage of 98% was selected. Short reads (38.2 million) were assembled into 19 contigs that were ordered within SeqMan Pro (Version 12, DNASTAR, Inc. Madison, WI, USA) using mate pair data. The first round assembling was then used as a template for a complete reassembly. Independently de novo assembled sequence data from other libraries were incorporated to proof read the first assembly and to maximize coverage and quality. Adjacent contigs, if possible, were merged. The alignment resulted in a three‐contig scaffold spanning the whole genome. Gaps were filled, and three contig were bridged and circled by Polymerase Chain Reaction (PCR) and Sanger sequencing. No contigs remained unassembled which might correspond to plasmids. The annotated MS14 genome, which has three chromosomes, is available from NCBI under the accession numbers: CP009743, CP009744, and CP009745.

### Other *Burkholderia* strains used for genome comparison

Seventeen previously sequenced *Burkholderia* strains were selected for comparative genome analysis based upon their characteristics and distinctive biological properties (Table 1). The selected species include MS14 and three PGPB *B. lata* 383, *B. ambifaria* AMMD, and *B. phytofirmans* PsJN (Coenye et al. 2001; Vanlaere et al. 2009; Weilharter et al. 2011), seven CF opportunistic pathogens and mammalian pathogens *B. thailandensis* E264, *B. mallei* ATCC 23344, *B. cenocepacia* J2315, *B. multivorans* ATCC 17616, *B. pseudomallei* 1026b, *B. pseudomallei* K96243, and *B. oklahomensis* EO147 (DeShazer et al. 2001; Biddick et al. 2003; Glass et al. 2006; Knappe et al. 2008; Holden et al. 2009). Plant‐pathogenic strains *B. gladioli* BSR3, which infects onions, rice, and iris (Seo et al. 2011), and *B. glumae* BGR1, which causes bacterial panicle blight on rice (Lim et al. 2009) were selected for comparison. Another five previously sequenced soil isolates including nitrogen‐fixing nodulator that were acquired from different habitats were also used: *B. cepacia* GG4, *B. phenoliruptrix* BR3459a, *B. phymatum* STM815, *B. xenovorans* LB400, and *B. vietnamiensis* G4 (Fries et al. 1997; Vandamme et al. 2002; Hong et al. 2012; de Oliveira Cunha et al. 2012).

**Table 1:** List of strains used in comparative analysis

Strain	Number of chromosomes	Number of plasmids	Size (Mb)	GC%	Gene	CDS	Source	Accession number
*B. contaminans* MS14	3	–	8.509	66.37	7582	7270	Cotton field, MS, USA	CP009743,CP009744,CP009745
*B. lata* 383	3	–	8.676	66.26	7823	7716	Forest soil, Trinidad and Tobago	NC_007510.1, NC_007511.1, NC_007509.1
*B. ambifaria* AMMD	3	1	7.529	66.79	6717	6610	Rhizosphere of healthy pea, Wisconsin, USA	NC_008390.1, NC_008391.1, NC_008392.1
*B. cenocepacia* J2315	3	1	8.056	66.92	7365	7116	CF patients, UK	NC_011000.1, NC_011001.1, NC_011002.1
*B. multivorans* ATCC 17616	3	1	7.009	66.69	6372	6258	Soil enriched with anthranilate, Berkeley, CA, USA	NC_010804.1, NC_010805.1, NC_010801.1
*B. gladioli* BSR3	2	4	9.052	67.41	7757	7411	Diseased rice sheath, South Korea.	NC_015381.1, NC_015376.1
*B. glumae* BGR1	2	4	7.285	67.93	6302	5773	Diseased Rice Grain, South Korea.	NC_012724.2, NC_012721.2
*B. mallei* ATCC 23344	2	–	5.836	68.52	5506	5022	Glanders patient, Myanmar	NC_006348.1, NC_006349.2
*B. oklahomensis* EO147	2	–	7.314	66.94	6357	6264	Wound infection, Georgia, USA	CP008726.1,CP008727.1
*B. phenoliruptrix* BR3459a	2	1	7.651	63.12	6605	6496	Root nodule of Mimosa flocculosa, South America.	NC_018695.1, NC_018672.1
*B. phymatum* STM815	2	2	8.677	62.28	7899	7496	Root nodule of Machaerium lunatum, French Guiana.	NC_010622.1, NC_010623.1
*B. phytofirmans* PsJN	2	1	8.215	62.32	7484	7241	Glomus vesiculiferum‐infected onion roots, plant‐beneficial bacterium	NC_010681.1, NC_010676.1
*B. pseudomallei* 1026b	2	–	7.231	68.16	6262	6070	Septicemic melioidosis patient, Thailand	NC_017831.1, NC_017832.1
*B. pseudomallei* K96243	2	–	7.248	68.05	5935	5727	Septicemic melioidosis patient, Thailand	NC_006350.1, NC_006351.1
*B. thailandensis* E264	2	–	6.724	67.65	5712	5632	Rice field. Thailand.	NC_007651.1, NC_007650.1
*B. vietnamiensis* G4	3	5	8.391	65.73	7861	7617	Soil, California, USA	NC_009256.1, NC_009255.1, NC_009254.1
*B. xenovorans* LB400	3	–	9.731	62.63	9043	8702	Contaminated soil, New York, USA	NC_007951.1, NC_007952.1, NC_007953.1
*B. cepacia* GG4	2	–	6.467	66.71	5903	5,825	Ginger rhizosphere, Malaysia	NC_018513.1, NC_018514.1

### Comparative analyses of *Burkholderia* genomes

Identification of putative protein‐encoding genes and annotation of *B. contaminans* MS14 whole genomes were performed by NCBI Prokaryotic Genome Automatic Annotation Pipeline (PGAAP) (Angiuoli et al. 2008), which was designed to annotate bacterial and archaeal genomes. Gene ontology (GO) analysis was performed by searching against protein database using BLAST2GO (Conesa et al. 2005), COG (http://www.ncbi.nlm.nih.gov/COG/) (Tatusov et al. 2003) and KEGG (Kyoto encyclopedia of genes and genomes; http://www.genome.jp/kegg/) (Kanehisa and Goto 2000). Progressive Mauve (Darling et al. 2010) was used to perform the multiple genome alignments. IslandViewer (Langille and Brinkman 2009) was used to predict and identify genomic islands (GIs) within the bacterial genomes with two different GIs prediction methods: SIGI‐HMM (Waack et al. 2006), which uses a hidden Markov model (HMM) (Baum and Petrie 1966) measuring codon usage for GIs prediction, and IslandPath‐DIMOB (Langille et al. 2008), which predicts GI based on identifying conserved regions across all genomes and unique regions to the query genome. Secondary metabolites‐ and antibiotics‐related genes were identified using antiSMASH (Medema et al. 2011). The average nucleotide identity (ANI) (Richter and Rossello‐Mora 2009) was calculated by a script developed by Kostas's lab (http://enve-omics.gatech.edu/), only the chromosome sequence of the *Burkholderia* strains were taken into account. Protein coding regions were visually compared using Easyfig (Sullivan et al. 2011) and genes were searched against the nonredundant database by BLASTx search. Protein sequence identity of 40% was used as the cutoff to distinguish between peptides of similar and nonsimilar structure (Rost 1999). BLASTn comparison of genomes was visualized by BRIG (Alikhan et al. 2011) and Circos (Krzywinski et al. 2009).

### Identification and comparison of genetic loci associated with siderophores and antimicrobial production, virulence factors and antibiotic resistance

The gene clusters required for production of siderophores and antimicrobial secondary metabolites were analyzed among the selected *Burkholderia* genomes. The gene clusters responsible for antimicrobial product biosynthesis were analyzed including occidiofungin, pyrrolnitrin, pyoluteorin, and lipopeptide AFC‐BC11 as mentioned previously. *Burkholderia rhizoxinica* produced antitumor product rhizoxin (Partida‐Martinez and Hertweck 2007) and *Burkholderia* spp produced spliceostatin (Eustaquio et al. 2014) were also included. The compared siderophores include pyochelin and ornibactin.

Pathogenesis‐related factors of *Burkholderia* species to both plants and mammals were compared. Plant‐toxic exopolysaccharide cepacian and toxins toxoflavin (Suzuki et al. 2004), hydrogen cyanide (HCN) (Ryall et al. 2008), and 2‐heptyl‐3‐hydroxy‐4(1H)‐quinolone (Diggle et al. 2006) were compared within the studied genomes as plant‐pathogenic agents. CF‐related O‐antigen of lipopolysaccharides pathogenic to mammals (Ortega et al. 2005) was also compared as it is associated with transmissible infections in CF patients.

The genes responsible for antibiotic resistance were analyzed within the researched genomes. First, four efflux pumps are compared: BpeAB‐OprB multidrug efflux pump that is responsible for the efflux of aminoglycosides gentamicin, streptomycin, and erythromycin (Chan et al. 2004), AmrAB–OprA efflux pump that is responsible for the extrude of aminoglycoside, macrolide, fluoroquinolones, and tetracyclines (Moore et al. 1999), BpeEF–OprC efflux pump that contributes to the resistance to chloramphenicol, fluoroquinolones, tetracyclines, and trimethoprim in clinical and environmental *B. pseudomallei* isolates (Podnecky et al. 2013), and a salicylate‐induced efflux pump that is associated with the resistance of chloramphenicol, trimethoprim, and ciprofloxacin (Nair et al. 2004). Biofilm formation and nonreplicating state alternation related genes were also compared: first, Cep Quorum‐sensing system‐related genes were selected for this system controls biofilm formation that could significantly reduce antimicrobial susceptibility (Huber et al. 2001). Second, arginine and pyruvate fermentation mechanism were analyzed for this mechanism are among those most highly induced in response to hypoxia under anaerobic conditions (Zuniga et al. 2002; Hamad et al. 2011), it is very likely that pathogenic *Burkholderia* bacteria utilize the same pathway for energy generation during respiratory stress in CF patients (Hamad et al. 2011). Moreover, beta‐lactamase‐induced enzymatic inactivation‐related genes were also analyzed due to their effects on ceftazidime and beta‐lactam drug clavulanic acid (Tribuddharat et al. 2003).

Virulence features associated genes that contributes to mammalian pathogenesis and opportunistic infections in CF were compared: Cable pili and the 22‐kilodalton adhesin biosnthesis genes that are required for *B. cenocepacia* binding and transmission, the *SodC* gene that encodes for a Cu^2+^ and Zn^2+^ containing periplasmic SOD that contributes to intracellular survival in CF patients, a zinc metalloprotease that may be involved in overall virulence of several Bcc strains(Corbett et al. 2003), the 31.7 kb *Burkholderia cepacia* virulence genomic island that harbors multiple virulence and metabolism‐associated genes, a melanin pigment that could aid the colonization and transmission of certain *B. cepacia* strains in CF patients (Zughaier et al. 1999), in addition, the VgrG‐5 protein that is required for type VI secretion system 5 to multinucleate giant cell formation (Schwarz et al. 2014).

## Results

### Genomic features of MS14

**Figure 1:**
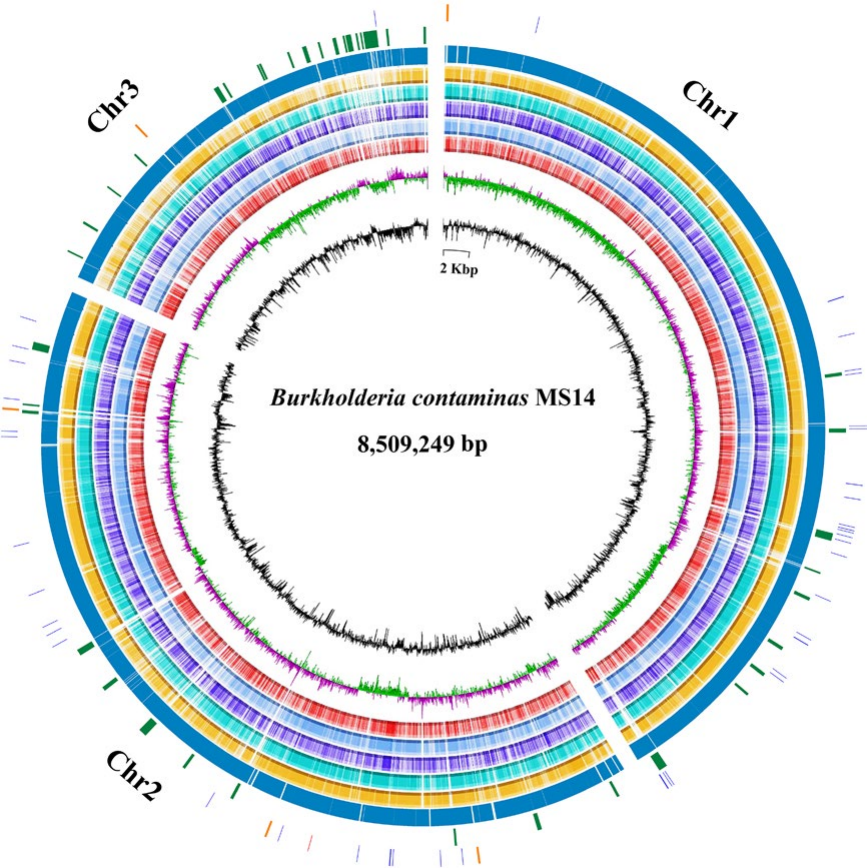
Circular representation of the *B. contaminans* MS14 genome in comparison with five sequenced *Burkholderia* whole genome. Rings from inside to outside: (1) GC content (black), (2) GC skew (purple and green), (3) BLAST comparison with *B. mallei* ATCC 23344 (red), (4) BLAST comparison with *B. cenocepacia* J2315 (aqua), (5) BLAST comparison with *B. glumae* BGR1 (slateblue), (6) BLAST comparison with *B. ambifaria* AMMD (cyan), (7) BLAST comparison with *B. lata* 383 (yellow), (8) Coding sequences of *B. contaminans* MS14 genome (dark blue), (9) Gene islands (dark green), (10) rRNA (yellow), tRNA (dark purple) and ncRNA (red). Figure generated by Circos with incorporated BLASTn result from BRIG (BLAST, Ring Image Generator).

The complete genome of *B. contaminans* MS14 consists of three circular chromosomes of 3,522,585, 3,358,952, and 1,627,712 bp with a GC content of 66.89%, 66.30%, and 65.50%, respectively. A total of 7813 genes were identified from the genome, of which 231 were pseudogenes or partial genes. The three replicons encode 3,020, 2,943, and 1,307 predicted coding DNA sequences (CDSs), 1 noncoding RNAs (ncRNAs), 5 rRNA operons, and 65 tRNA loci. Forty‐nine genomic islands ranging from 4 kbp to 29 kbp were also identified by Islandviewer throughout the MS14 genome of which 22 genomic islands were identified from chromosome 3 (Table S1). Majority of the genomic islands genes encode hypothetical proteins. A summary of *B. contaminans* MS14 genome features are provided in Table 2 and the circular chromosomes are provided in Figure 1.

**Table 2:** Chromosome statistics of *Burkholderia contaminans* MS14

Feature	Chromosome 1	Chromosome 2	Chromosome 3	Total
Size	3,522,585 bp	3,358,952 bp	1,627,712 bp	8,509,249 bp
Genes	3130	3,049	1403	7582
CDS	3020	2,943	1307	7270
Pseudogenes	67	73	91	231
rRNAs	1	3	1	5
tRNAs	40	23	2	65
ncRNA	0	1	0	1
G+C content	66.89%	66.30%	65.50%	66.40%

An insight of *B. contaminans* MS14 genome indicated multiple predicted antibiotic‐ and antimicrobial‐related secondary metabolites biosynthetic gene loci by antiSMASH (Table S2), those include five terpene and two bacteriocin biosynthesis gene clusters. Two NRPS genes NL30_14890 and NL30_14895 on chromosome one share 91% and 90% nucleotide sequence similarity with *orbJ* and *orbI* NRPS genes in the ornibactin biosynthetic gene cluster (Agnoli et al. 2006), respectively. On the chromosome three, the genes NL30_32800, NL30_32795, NL30_32790, and NL30_32785 share an overall 90% nucleotide sequence similarity to the *prnABCD* genes in pyrrolnitrin biosynthetic gene cluster (Costa et al. 2009). In addition, two PKS genes NL30_36200 and NL30_36225 share a highest 69% and 66% amino acid sequence similarity to any published data. Strain MS14 is likely to produce a novel bioactive compound via those two PKS genes.

Blast research of *B. contaminans* MS14 genome against *B. lata* 383, *B. ambifaria* AMMD, *B. glumae* BGR1, *B. cenocepacia* J2315, and *B. mallei* ATCC 23344 genome revealed multiple unique gene regions, which were only found in the MS14 genome (Fig. 1). The BLASTn atlas showed that MS14 chromosome presents a large similarity with the PGPB *B. lata* 383, there is no surprise considering they belonged to taxon K of the Bcc prior to their reassignment as two different species. However, 5% coding genes present in MS14 chromosome did not share significant homology with any other species used in comparison. The BLASTn atlas also indicated that MS14 chromosome 3 is more diverse than the MS14 chromosomes 1 and 2, harboring more unique gene regions that do not share significant similarity with those in other genomes being compared. Moreover, MS14 chromosome 3 harbors majority of gene islands identified within its genome.

**Figure 2:**
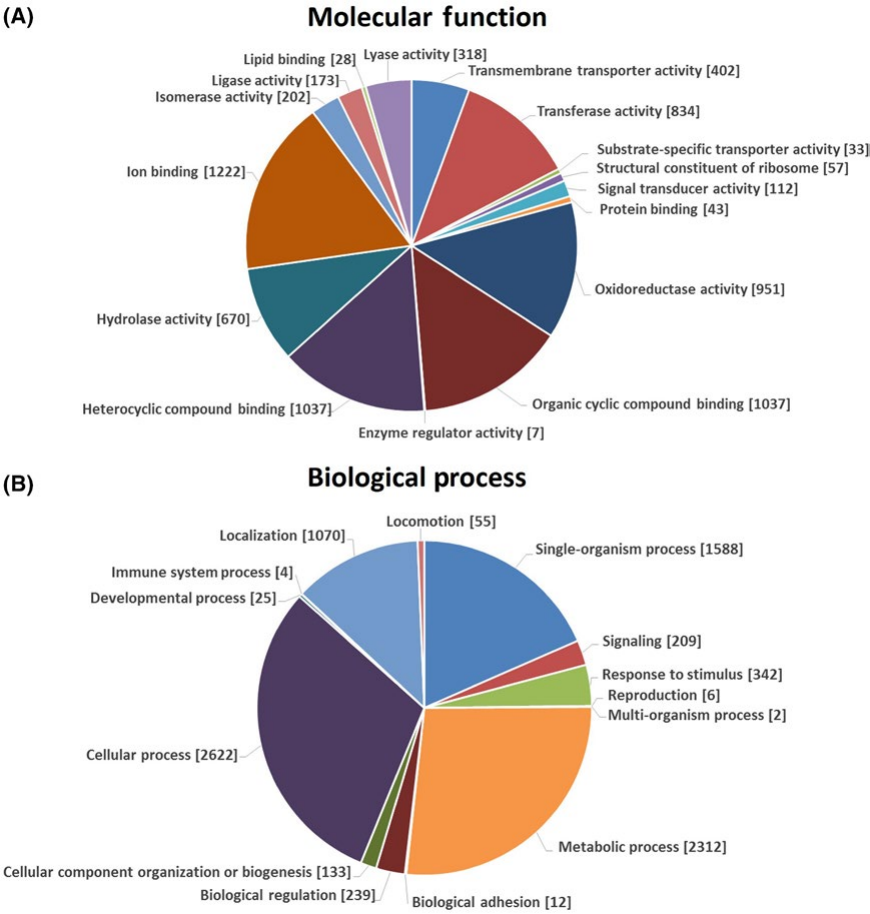
Gene ontology (GO) analyses of *B. contaminans* MS14 genome. GO analysis of *B. contaminans* MS14 genome corresponding to 7,582 genes as for their predicted involvement in molecular functions (A) and biological processes (B). Data are presented as level 3 GO categorization for molecular function and level 2 GO categorization for biological process. Classified gene objects are depicted as gene numbers (in brackets).

Gene ontology annotations were added to MS14 genes to conduct gene analysis in population according to analyzed transcripts (Ashburner et al. 2000). GO term distribution describing molecular function and biological process is shown in Figure 2. Besides the basic cell functions, GO terms connected to heterocyclic compound binding and organic cyclic compound binding are well represented in MS14 genome, that can selectively and noncovalently interact with heterocyclic compound and organic cyclic compound, of which many bioactive secondary metabolic are derived from. In addition, GO terms associated with ion binding, hydrolase activity, oxidoreductase, and transferase activity are also represented in large percentage in MS14 genome. On the other side, more than a quarter of the biological process GO terms are connected to the metabolic process. Those data indicate that, like some *Burkholderia* strains (Bartell et al. 2014), strain MS14 is very versatile from the metabolic standpoint.

### Phylogenetic relationships of sequenced *Burkholderia* species

Phylogenetic relationships of sequenced *Burkholderia* species were analyzed. Strains with ANI values greater than 96%, which equate to a DNA–DNA hybridization value of 70%, are considered to be the same species (Richter and Rossello‐Mora 2009). Strains with ANI value greater than 90% are considered to have high genome relatedness. Strains with ANI value lower than 90% are considered to have divergent genomes (Konstantinidis et al. 2006). As shown in Table 3, strain MS14 has the greatest nucleotide identity similarity to *B. lata* 383, which is also a novel species within *Burkholderia* taxon K (Vanlaere et al. 2009). Soil isolate *B. cepacia* GG4 is most closely related to the PGPB strains *B. ambifaria* AMMD. The pathogenic *B. cenocepacia* J2315 is also closely related to PGPB strain MS14, 383, AMMD, soil isolates GG4 and G4 than to other pathogenic species. Soil isolates GG4 and G4 have high genome relatedness to pathogenic *B. multivorans* ATCC 17616. Plant‐pathogenic *B. gladioli* BSR3 and *B. glumae* BGR1 only showed limited gene relatedness between them due to the 2 Mb genome size difference, even though they share a similar host range. These ANI data suggest that genome sequences of the PGPB isolates are different from some virulent strains such as *B. pseudomallei* 1026b, *B. pseudomallei* K96243, and *B. mallei* ATCC 23344, which have very close phylogenetic relationship between them (ANI > 99%).

**Table 3:**
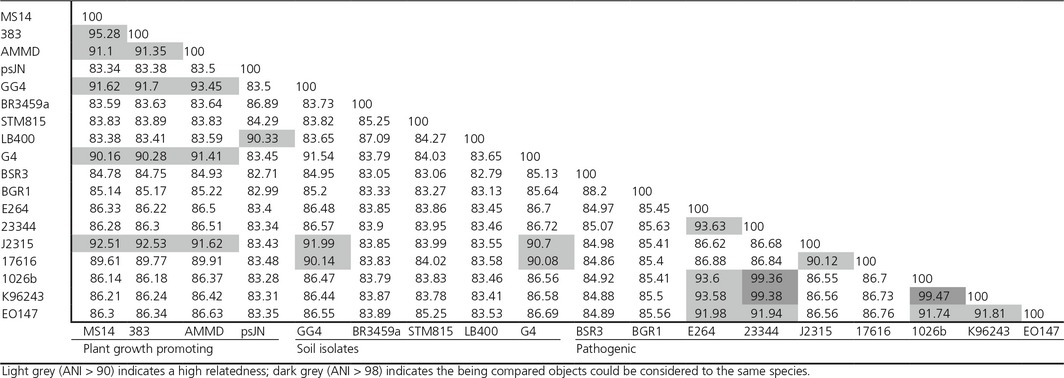
Average Nucleotide Identity (ANI) pairwise comparisons among sequenced Burkholderia strains

### Production of siderophores and antimicrobial compounds

**Figure 3:**
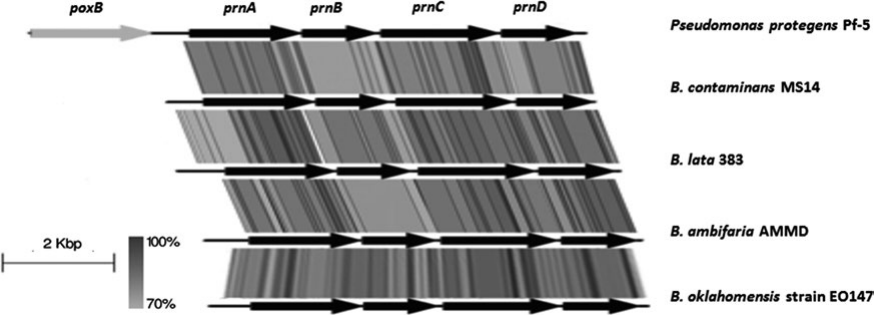
Pyrrolnitrin biosynthesis locus genetics. Figure showing the Pyrrolnitrin biosynthesis locus identified in analyzed Burkholderia genomes. The CDS marked in black are the *prnABCD* operon, which is highly conserved in the four *Burkholderia* strains showing in the figure. Figure was generated by Easyfig.

The comparison of antibiotics production‐related gene loci among the *Burkholderia* species are shown in Table 4. Only the core biosynthesis genes are being compared, as indicated in the table. Intact occidiofungin biosynthesis gene is only present in the AMMD and MS14. Several *Burkholderia cepacia* species were reported to produce pyrrolnitrin. As expected, the PGPB MS14, 383, AMMD, and *B. oklahomensis* EO147 all harbor the intact biosynthesis gene *prnABCD* as a gene cluster (Fig. 3). However, the pyrrolnitrin biosynthesis locus was not found in *B.phytofirmans* PsJN. Rhizoxin's biosynthesis gene *RhiABCDE* and spliceostatin biosynthesis gene clusters were not found in the *Burkholderia* genomes. Antifungal compound pyoluteorin biosynthesis genes *pltB and pltC* were not found in the analyzed genomes. Intact siderophore pyochelin biosynthesis *pch* gene cluster and ornibactin biosynthesis *orb* gene *orbI* and *orbJ* are widely possessed among the studied genomes compared to the lipopeptide AFC‐BC11, whose biosynthesis genes *afcBACD* are only harbored by MS14, 383, AMMD, and pathogenic *B. cenocepacia* J2315. None of the antimicrobial biosynthesis genes being studied were identified from soil isolate *B. phenoliruptrix* BR3459a and plant‐pathogen bacteria *B. gladioli* BSR3, and *B. glumae* BGR1.

**Table 4:** Distribution of antibiotic and virulence compound symbiotic loci of *Burkholderia* strains

Name		Biosynthesis Gene Homologs	PGPB				Soil isolates					Pathogenic								
			383	MS14	AMMD	psJN	GG4	BR3459a	STM815	LB400	G4	BSR3	BGR1	E264	23344	J2315	17616	1026b	K96243	EO147
Antibiotic and siderophore	Occidiofungin	ocfD‐ocfJ	**‐**	**+**	**+**	**−**	**−**	**−**	**−**	**−**	**−**	**−**	**−**	**−**	**−**	**−**	**−**	**−**	**−**	**−**
	Pyrrolnitrin	prnA‐prnD	**+**	**+**	**+**	**−**	**−**	**−**	**−**	**−**	**−**	**−**	**−**	**−**	**−**	**−**	**−**	**−**	**−**	**+**
	Rhizoxin	rhiA‐rhiF	**−**	**−**	**−**	**−**	**−**	**−**	**−**	**−**	**−**	**−**	**−**	**−**	**−**	**−**	**−**	**‐**	**−−**	**−**
	Spliceostatin	fr9C‐fr9I	**−**	**−**	**−**	**−**	**−**	**−**	**−**	**−**	**−**	**−**	**−**	**−**	**−**	**−**	**−**	**−**	**−**	**−**
	Pyoluteorin	pltB, pltC	**−**	**−**	**−**	**−**	**−**	**−**	**−**	**−**	**−**	**−**	**−**	**−**	**−**	**−**	**−**	**−**	**−**	**−**
	Pyochelin	pchR, pchD‐pchA	**+**	**−**	**−**	**−**	**−**	**−**	**−**	**−**	**−**	**−**	**−**	**+**	**−**	**+**	**−**	**+**	**+**	**−**
	Ornibactin	orbE, orbI, orbJ	**+**	**+**	**+**	**+**	**+**	**−**	**+**	**+**	**+**	**−**	**−**	**+**	**+**	**+**	**+**	**+**	**+**	**+**
	AFC**‐**BC11	afcB, afcA, afcC, afcD	**+**	**+**	**+**	**−**	**−**	**−**	**−**	**−**	**−**	**−**	**−**	**−**	**−**	**+**	**−**	**−**	**−**	**−**
Virulence metabolics	Cepacian	bceA‐bceK, bceN‐bceT	**+**	**+**	**+**	**+**	**+**	**+**	**+**	**+**	**+**	**+**	**+**	**+**	**+**	**+**	**+**	**+**	**+**	**+**
	Toxoflavin	toxR, toxA‐toxE	**−**	**−**	**−**	**−**	**−**	**−**	**−**	**−**	**−**	**+**	**+**	**−**	**−**	**−**	**−**	**−**	**−**	**−**
	Hydrogen Cyanide	hcnA‐hcnC	**−**	**−**	**+**	**−**	**+**	**−**	**+**	**−**	**+**	**+**	**+**	**+**	**+**	**+**	**+**	**+**	**+**	**+**
	2‐heptyl‐3‐hydroxy‐4(1H)‐quinolone	pqsA‐pqsE	**−**	**−**	**−**	**−**	**−**	**−**	**−**	**−**	**−**	**−**	**−**	**+**	**+**	**−**	**+**	**+**	**+**	**+**

### Virulent secondary metabolites production

Plant‐virulent secondary metabolites‐related loci are compared and shown in Table 4. The cepacian biosynthesis genes are possessed among all *Burkholderia* genomes. Only the two plant‐pathogen BSR3 and BGR1 harbors toxoflavin biosynthesis gene *toxR* and *toxA‐toxE* homologs. Conversely, HCN and 2‐heptyl‐3‐hydroxy‐4(1H)‐quinolone biosynthesis gene *pqsA‐pqsE* homologs are more commonly possessed by the studied genomes. AMMD has hydrogen cyanide gene cluster identified, which is not present in the PGPB strains MS14 and 383. The overall data show MS14, 383, AMMD, and other environmental isolates have very few toxin biosynthesis genes in their genome compared to those of the genomes of the plant‐pathogenic species.

**Figure 4:**
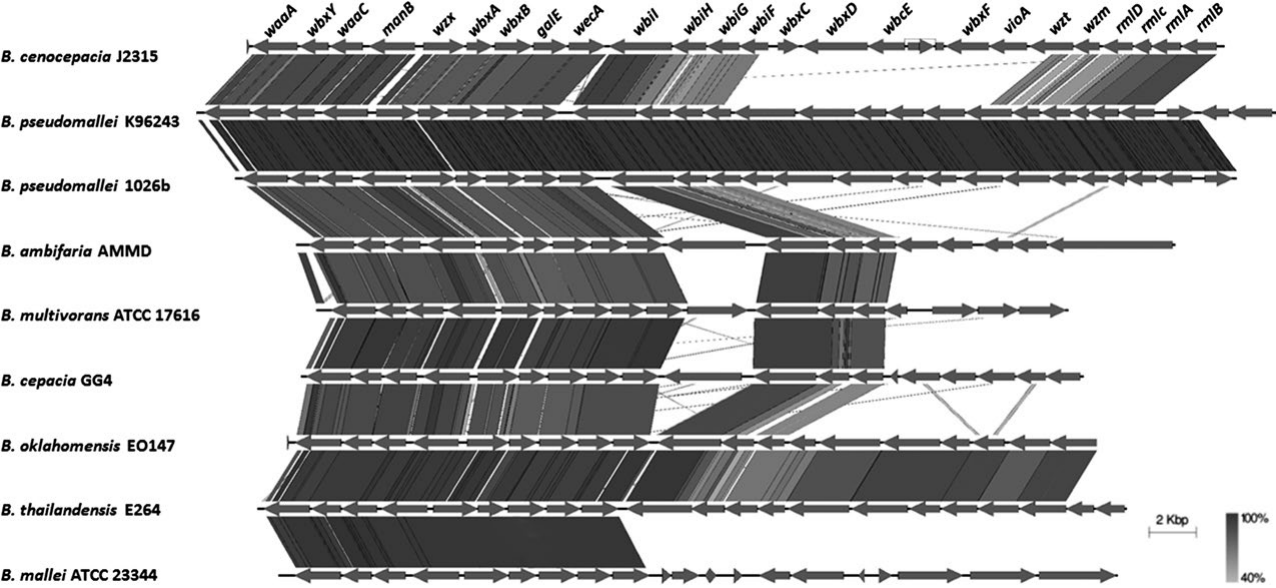
Cystic fibrosis (CF)‐related O‐antigen biosynthesis locus genetics. Figure showing a 29 kb gene region of *B. cenocepacia* J2315 containing 24 genes responsible for O‐antigen biosynthesis and lipid A‐core component.

Human pathogenic CF‐related O‐antigen biosynthesis gene cluster of selected *Burkholderia* species were compared, the O‐antigen biosynthesis locus genetics are shown in Figure 4. The 29 kb gene region of *B. cenocepacia* J2315 containing 24 genes responsible for O‐antigen biosynthesis and lipid A‐core component was compared to the other 17 *Burkholderia* species. The homologs of first 13 genes, from *waaA* to *wbiF* that are required for the biosynthesis of lipid A‐core component, assembly initiation of the O antigen subunits and translocation of O‐antigen subunit across membrane*,* were identified from the genomes of all the Bcc species analyzed in this study. The homologs of the *wbxCDEF* genes that are also required for O‐antigen biosynthesis were identified from K96243, 1026b, AMMD, 17616, GG4, and EO147 genome (not shown in the BLAST region), but not identified from strains E264 and 23344. Possessing large amount of genes within O‐antigen cluster indicates a high possibility of a strain's virulence (Ortega et al. 2005).

### Virulence features

Pathogenic *Burkholderia* species including CF opportunistic isolates have more key virulent genes identified than nonpathogenic species (Table 5). Strain J2315 harbors intact cable pili and the 22‐kilodalton adhesin biosynthesis gene, strain 17,616 harbors intact adhesin biosynthesis gene cluster, but lacks the cable pili biosynthesis gene cluster. Moreover, lack of cable pili biosynthesis genes in other *Burkholderia* species indicates their inability to attach to the host cell to initiate infection. The periplasmic superoxide dismutase *SodC* gene (Keith and Valvano 2007) is commonly present among Bcc species indicating their self‐protection ability. Zinc metalloprotease biosynthesis genes were found in the PGPB genomes; however, there is no evidence showing a direct relationship between the production of zinc metalloprotease and virulence. The 31.7 kb *Burkholderia cepacia* virulence genomic island is only harbored by strain J2315. The lack of this genomic island indicates a significant decrease in the pathogenicity potential in other *Burkholderia* species. The biosynthesis gene of melanin had been identified from all analyzed *Burkholderia* genomes. VgrG‐5 protein biosynthesis gene is present in the pathogenic strains E264, 23344, 1026b, K96243, EO147.

**Table 5:** Distribution of antibiotic resistance and pathogenic symbiotic loci of *Burkholderia* strains

Name		PGPB				Soil strains					Pathogenic strains								
		383	MS14	AMMD	psJN	GG4	BR3459a	STM815	LB400	G4	BSR3	BGR1	E264	23344	J2315	17616	1026b	K96243	EO147
*Antibiotic resistance*	*BpeAB‐OprB* Multidrug Efflux Pump	**+**	**+**	**+**	**+**	**+**	**+**	**+**	**+**	**+**	**+**	**+**	**+**	**+**	**+**	**+**	**+**	**+**	**+**
	*AmrAB–OprA* Multidrug Efflux Pump	**+**	**+**	**+**	**−**	**+**	**−**	**+**	**+**	**+**	**+**	**+**	**+**	**+**	**+**	**+**	**+**	**+**	**+**
	*BpeEF‐OprC* Multidrug Efflux Pump	**+**	**+**	**+**	**+**	**+**	**+**	**+**	**+**	**+**	**+**	**+**	**+**	**+**	**+**	**+**	**+**	**+**	**+**
	*Salicylate‐Induced* Antibiotic Efflux Pump	**+**	**+**	**+**	**+**	**+**	**+**	**+**	**+**	**+**	**+**	**+**	**+**	**+**	**+**	**+**	**+**	**+**	**+**
	*Cep* Quorum‐sensing System	**+**	**+**	**+**	**−**	**+**	**−**	**−**	**−**	**+**	**+**	**+**	**+**	**+**	**+**	**+**	**+**	**+**	**+**
	Arginine and Pyruvate Fermentation	**−**	**−**	**−**	**+**	**−**	**−**	**+**	**+**	**−**	**−**	**−**	**+**	**+**	**−**	**−**	**+**	**+**	**+**
	Beta‐Lactamase	**+**	**+**	**+**	**−**	**+**	**−**	**+**	**−**	**+**	**+**	**+**	**+**	**+**	**+**	**+**	**+**	**+**	**+**
	Porins	**+**	**+**	**+**	**+**	**+**	**+**	**+**	**+**	**+**	**+**	**+**	**+**	**+**	**+**	**+**	**+**	**+**	**+**
*Pathogenic symbiotic*	Cable Pili	**−**	**−**	**−**	**−**	**−**	**−**	**−**	**−**	**−**	**−**	**−**	**−**	**−**	**+**	**−**	**−**	**−**	**−**
	22‐Kilodalton Adhesin	**−**	**−**	**−**	**−**	**−**	**−**	**−**	**−**	**−**	**−**	**−**	**−**	**−**	**+**	**+**	**−**	**−**	**−**
	Periplasmic Superoxide Dismutase SodC	**+**	**+**	**+**	**+**	**+**	**+**	**+**	**+**	**+**	**+**	**+**	**+**	**+**	**+**	**+**	**+**	**+**	**+**
	Zinc Metalloprotease	**+**	**+**	**+**	**−**	**−**	**−**	**−**	**−**	**−**	**−**	**−**	**+**	**+**	**+**	**−**	**+**	**+**	**+**
	Burkholderia cepacia Virulence Genomic Island	**−**	**−**	**−**	**−**	**−**	**−**	**−**	**−**	**−**	**−**	**−**	**−**	**−**	**+**	**−**	**−**	**−**	**−**
	Melanin	**+**	**+**	**+**	**+**	**+**	**+**	**+**	**+**	**+**	**+**	**+**	**+**	**+**	**+**	**+**	**+**	**+**	**+**
	Type VI Secretion System‐Exported Protein VgrG‐5	**−**	**−**	**−**	**−**	**−**	**−**	**−**	**−**	**−**	**−**	**−**	**+**	**+**	**−**	**−**	**+**	**+**	**+**

### Antibiotic resistance

Antibiotic resistance is a common feature among studied *Burkholderia* (Table 5). First, our results show that the four well‐studied efflux pumps (AmrAB–OprA efflux pump, BpeAB‐OprB multidrug efflux pump, BpeEF–OprC efflux pump, and salicylate‐induced efflux pump) are commonly present among the sequenced genomes, however, the AmrAB–OprA efflux pump was not identified from genome of strains psJN and BR3459a. Cep quorum‐sensing system is also commonly identified in the sequenced genomes. This data indicated that the majority of the sequenced *Burkholderia* species being analyzed have biofilm formation potential. However, the exceptions were psJN and the soil isolates BR3459a, STM815, and LB400. Arginine and pyruvate fermentation‐related genes were identified from PGPB strain psJN, soil isolates STM815, LB400 and pathogenic strains E264, 23344, 1026b, K96243, and EO147. The biosynthesis of beta‐lactamase gene was not identified from psJN, BR3459a, and GG4. Taking all into account, the results provide genetic evidence to understand *Burkholderia* species possess multiple antibiotic resistance mechanisms to adapt and to survival different the environment. Overall, pathogenic *Burkholderia* species possess a majority of both antibiotic‐resistant genes and virulence‐related genes. The PGPB strains, on the other hand, have the majority of the antibiotic‐resistant‐related genes, but lack the key virulence‐related genes like the biosynthesis genes for cable pili, adhesin, and the VgrG‐5 protein.

## Discussion

*B. contaminans* MS14 is a versatile environmental isolate that shows significant antifungal and antibacterial activity. By investigating MS14′s genome, we found loci contributing to biosynthesis of several antimicrobial agents, which may partially explain its plant growth‐promoting activity. There are multiple gene regions related to antimicrobial production or antagonistic activity. The wide spectrum of antibiotic biosynthesis genes possessed by MS14 may contribute to its survivability when competing with other soil‐born microorganisms.

Genome comparative analysis shows significant horizontal gene transfers occurred during the evolution of MS14. The MS14 genome contains 49 genomic islands that are absent in other studied *B. contaminans* genomes. One genomic island on chromosome 3 encodes a putative‐exported protein that has a 58% amino acid identify to the type VI secretion protein Rhs of *Ralstonia* sp. GA3‐3 (NCBI Reference Sequence: WP_037024668.1) (Pearce et al. 2013). This protein is predicted to facilitate the exclusion of molecules from the cell wall and is possibly involved in the antibiotic resistance features (Filloux et al. 2008). The acquisition and exchange of mobile genetic elements in addition to GIs has contributed to the overall genome plasticity as well as MS14 antagonistic activities. Presumably, the integration of new biosynthetic pathways has made the bacterial strain more adaptated to various ecological niches. The contribution of the other GIs to survival of MS14 remains unknown, since many of the coding genes in the GIs code for hypothetical proteins.

The average nucleotide identity is one of the most robust measurements of genomic relatedness between strains (Kim et al. 2014). The ANI data show that the nonpathogenic and pathogenic isolates are clustered together, respectively. These results confirm that distinct lineages exist among *Burkholderia* species (Angus et al. 2014). The ANI values between *B. pseudomallei* 1026b, *B. pseudomallei* K96243, and *B. mallei* ATCC 23344 were previously shown to be all above 99%. Therefore, these three isolates can be considered the same species according to Konstantinidis et al. (Konstantinidis et al. 2006). However, progressive Mauve results on chromosome 1 indicate significant gene shuffling and rearrangement between these three species, suggesting a limited gene synteny between the isolates that have very high ANI values (Fig. S1). What is learned from these observations is that considerable gene rearrangement can occur between two species that have more than 99% ANI. Interestingly, pathogenic *B. cenocepacia* J2315 has a high genome relatedness to PGPB MS14, 383, and AMMD, even the following genomic analysis shows J2315 harbors almost all key virulent‐related genes, which are not present in the PGPB species. *Burkholderia* species can have very high flexibility and mosaicism among their genomes. These results support the notion that conventional classification of *Burkholderia* species is not able to distinguish between pathogenic and nonpathogenic species and that a whole genome comparison as was conducted with MS14 is needed to make this distinction.

MS14, PGPB 383 and AMMD have a wide range of common antimicrobial biosynthesis gene clusters within their genomes, supporting the possible production of multiple antimicrobial agents that contribute to their plant growth‐promoting activity by inhibiting soil‐borne plant pathogens, including pathogenic fungi and bacteria. Those antimicrobial agents include bactericidal agents like pyrrolnitrin and bacteriostatic siderophores. Occidiofungin is highly effective in killing pathogenic fungi by altering cell wall integrity or by inducing apoptosis (Lu et al. 2009; Emrick et al. 2013), however, its biosynthesis gene cluster was not found in the closely related 383. Alternatively, another pyochelin biosynthesis genes were found in 383 that is not present in MS14 genome. These results show that the PGPB strains have multiple mechanisms for inhibiting competing microorganism that increase their survivability.

Euan L.S. Thomson and Jonathan J. Dennis reported that *B. vietnamiensis* DBO1 harbors an occidiofungin gene cluster homolog that is related to hemolytic activity and virulence (Thomson and Dennis 2012). However, two key polyketide biosynthesis genes 6477 and 6478 presented in DBO1 that are required for full virulence were not found in the MS14 genome. According to the author, the disruption of either of those two polyketide synthase genes led to a complete loss of human erythrocytes hemolytic activity. Conversely, hemolytic activity can be utilized in beneficial way. For example, hemolytic bacterium *Pseudomonas entomophila* had been utilized as entomopathogenic bacterium in killing insects to protect plants (Vallet‐Gely et al. 2010). Moreover, hemolytic activity has not been reported to increase a strain's chance of being an opportunistic microorganism. According to the same research data, only one out of 30 CF *Burkholderia* isolates have detectable hemolytic activity, yet regular soil/water *Burkholderia* isolates have shown moderate to high hemolytic activity. This observation indicates that hemolytic activity is not an important determinant for virulence of Burkholderia species and that the potential for hemolytic activity does not increase the possibility of MS14's chance of being a CF opportunistic bacterium.

Toxin biosynthesis clusters were mainly identified from pathogenic *Burkholderia* species, as expected. For example, toxoflavin biosynthesis gene cluster was identified from the plant‐pathogen species BSR3 and BGR1, this result further confirmed their pathogenicity in inducing bacterial blight in many field crops (Lim et al. 2009; Seo et al. 2011). The HCN is extremely toxic to both plants and human beings (http://pubchem.ncbi.nlm.nih.gov/compound/hydrogen_cyanide#section=Top; http://www.cyanidecode.org/cyanide-facts/environmental-health-effects). The biosynthesis genes for HCN production are present in all pathogenic species. Additionally, all pathogenic species in this study have the O‐antigen biosynthesis‐related genes associated with CF patient infection. It is not surprising that pathogenic isolates have more toxin‐producing genes. However, PGPB AMMD strain also has the CF‐related O‐antigen biosynthesis genes and hydrogen cyanide biosynthesis genes. This observation shows that the Bcc strain AMMD, which is effective at controlling plant‐pathogenic fungi, may contribute to CF infections. Given the presence of such virulence factors should raise concern for its use as a PGPB in agriculture. Whereas, MS14 and 383 only have the cepacian *bce‐I* gene cluster. Since cepacian is not required for the initiation of biofilm formation and only facilitates bacterial persistence (Cunha et al. 2004; Ferreira et al. 2010), the production of cepacian is not a major virulence determinant.

Antibiotic resistance is a very common feature among *Burkholderia* species. Possessing multiple efflux pumps facilitates viability of bacterial cells in different ecological niches. MS14 and 383 do not have arginine and pyruvate fermentation mechanism‐related genes that are crucial in cell energy generation under low oxygen conditions. The species that possess arginine and pyruvate fermentation are more capable of surviving in low oxygen environments, such as the environment found in mucus of CF patients' respiratory system. The cells remain latent in low oxygen environments making them more difficult to be killed by antibiotics (Hamad et al. 2011). Lack of arginine and pyruvate fermentation‐related genes in MS14 and 383 genomes indicates that both of these strains are not likely chronic opportunistic microorganisms.

PGPB and environmental isolates lack the key pathogenic features, such as cable pili formation and adhesin secretion activity. These are necessary for initiating and causing a CF opportunistic infection. Type VI secretion systems that have been reported to be related to animal's melioidosis (Burtnick et al. 2011) have been identified from all researched *Burkholderia* genomes. However, the VgrG‐5 protein, which is required for type VI secretion system 5 to induce multinucleate giant cell formation and full virulence, has its biosynthesis genes only identified from most pathogenic species. Melanin aids in environmental survival, however, the relationship between the production of melanin and the increase in virulence is unknown (Keith et al. 2007). The virulence feature comparison shows that PGPB and environmental isolates have less virulence‐related genes in their genomes, which indicates that PGPB characterized in this study pose less risk of causing an infection when compared to the pathogenic and CF opportunistic species.

The studied *Burkholderia* species can be separated into a virulent group and a less‐virulent group based on the data summarized above. The first group includes opportunistic pathogens, mammalian pathogens, and plant pathogens. This group is highly problematic in causing human or plant disease due to multiple virulence‐related genes identified from their genomes. The less‐virulent group includes plant growth‐promoting species, nitrogen‐fixing nodulators, and other environmental isolates that were used in the genomic analysis. The analysis of virulence traits in the genomes of the PGPB further confirmed that the plant‐associated symbiotic *Burkholderia* species lack hallmark strategies required in mammalian pathogenesis as described by Angus et al. (Angus et al. 2014). Those studied *Burkholderia* species are highly unlikely to be pathogenic to either plants or mammals based on the functional gene analysis. Whole genomic analysis approach as described in our study, should be prompted to identify the virulence level of a *Burkholderia* isolate.

## Conclusion

*B. contaminans* MS14 has three chromosomes with a genome size of 8.5 Mb. Multiple antibiotic and bacteriocin biosynthesis genes contribute to its overall antimicrobial activity. BLASTn result of MS14 against other *Burkholderia* genomes reveals 362 unique coding genes that are not present in other researched genomes. Fifty‐one percent of the unique genes encode protein products with unknown function, 18% of the unique genes encode for metabolic and secretion system‐related proteins. ANI data indicates MS14 is closely related to *B. lata* 383 with a 95.28% ANI score, and the ANI data also demonstrates the diversity of *Burkholderia* species genomes. However, significant syntenic gene rearrangement can occur between two very closely related genomes, for example, between *B. pseudomallei* K96243 and *B. pseudomallei* 1026b, which have a more than 99% ANI score. The secondary metabolites biosynthesis genes and virulence‐associated loci were compared among *Burkholderia* species. These comparisons provide an insight of distinguishing between pathogenic and nonpathogenic *Burkholderia* species.

MS14 has biosynthesis gene cluster identified for antibiotic occidiofungin, pyrrolnitrin, ornibactin, and AFC‐BC11. Other strains, such as PGPB 383 and AMMD also have similar antibiotic biosynthesis gene clusters identified from their genomes. Conversely, MS14 does not have gene homologs for the production of virulent secondary metabolite toxoflavin, hydrogen cyanide, or 2‐heptyl‐3‐hydroxy‐4(1H)‐quinolone, which are commonly produced by plant‐pathogenic *Burkholderia* species. Besides, MS14 does not harbor gene loci that are related to key virulence features possessed by human pathogenic Bcc species. For example, MS14 does not have biosynthesis gene homologs for cable pili, 22‐Kilodalton adhesion, virulent protein VgrG‐5, or the intact *Burkholderia cepacia* virulence genomic island that harbors BCESM. These results indicate that MS14 has a low virulent potential and could be used to separate MS14 from the pathogenic *Burkholderia* species. Consequently, soil isolates could also be distinguished from pathogenic strains by the analysis of established virulence factors and feature genes.

## Conflict of Interests

The authors declare no competing interests.

## Ethics Statement

This research did not involve any human or animal subjects, materials, or data and therefore did not require any ethics oversight or approval in these respects.

## Supporting information


**Table S1**. Gene islands of *Burkholderia contaminans* MS14.
**Table S2.** Secondary metabolites and antibiotics prediction of Burkholderia contaminans MS14.
**Figure S1.** Synteny of *B. mallei* ATCC 23344, *B. pseudomallei* K96243 and *B. pseudomallei* 1026b chromosome 1.
