# Supplementary material for: Comparative genome‐wide analysis reveals that Burkholderia contaminans MS14 possesses multiple antimicrobial biosynthesis genes but not major genetic loci required for pathogenesis
Source: Microbiologyopen. 2016 Jan 14;5(3):353–69. doi: 10.1002/mbo3.333 (PMC4905989; doi:10.1002/mbo3.333)
Supplement: Supplementary file 1 — Table S1. Gene islands of Burkholderia contaminans MS14. Table S2. Secondary metabolites and antibiotics prediction of Burkholderia contaminans MS14. Figure S1. Synteny of B. mallei ATCC 23344, B. pseudomallei K96243 and B. pseudomallei 1026b chromosome 1. [file MBO3-5-353-s001.docx]

| Table S1. Gene islands of *Burkholderia contaminans* MS14 | | | |
| --- | --- | --- | --- |
|  | Start (bp) | End (bp) | Size (bp) |
| Chromosome 1 | 1,901,874 | 1,911,806 | 9,932 |
|  | 2,091,575 | 2,104,192 | 12,617 |
|  | 2,448,109 | 2,472,973 | 24,864 |
|  | 2,454,931 | 2,467,588 | 12,657 |
|  | 2,585,967 | 2,590,542 | 4,575 |
|  | 2,678,230 | 2,687,979 | 9,749 |
|  | 2,868,720 | 2,879,187 | 10,467 |
|  | 2,976,432 | 2,986,943 | 10,511 |
|  | 3,064,366 | 3,071,669 | 7,303 |
|  | 3,432,906 | 3,461,804 | 28,898 |
|  | 3,434,508 | 3,444,193 | 9,685 |
|  | 3,446,070 | 3,459,731 | 13,661 |
| Chromosome 2 | 45,853 | 53,600 | 7,747 |
|  | 328,932 | 342,001 | 13,069 |
|  | 330,055 | 336,094 | 6,039 |
|  | 619,335 | 625,258 | 5,923 |
|  | 1,397,877 | 1,406,627 | 8,750 |
|  | 1,589,491 | 1,598,229 | 8,738 |
|  | 1,762,837 | 1,789,899 | 27,062 |
|  | 1,769,896 | 1,774,439 | 4,543 |
|  | 1,777,590 | 1,782,855 | 5,265 |
|  | 1,966,943 | 1,979,883 | 12,940 |
|  | 2,111,739 | 2,132,117 | 20,378 |
|  | 2,966,250 | 2,975,226 | 8,976 |
|  | 2,993,306 | 2,997,669 | 4,363 |
|  | 3,183,581 | 3,208,750 | 25,169 |
|  | 3,191,886 | 3,198,166 | 6,280 |
| Chromosome 3 | 122,317 | 126,399 | 4,082 |
|  | 229,650 | 236,433 | 6,783 |
|  | 375,976 | 380,290 | 4,314 |
|  | 516,919 | 522,413 | 5,494 |
|  | 866,117 | 882,546 | 16,429 |
|  | 900,733 | 906,564 | 5,831 |
|  | 1,031,178 | 1,036,272 | 5,094 |
|  | 1,039,681 | 1,045,537 | 5,856 |
|  | 1,141,808 | 1,147,600 | 5,792 |
|  | 1,195,257 | 1,208,241 | 12,984 |
|  | 1,249,881 | 1,255,495 | 5,614 |
|  | 1,301,120 | 1,312,049 | 10,929 |
|  | 1,336,666 | 1,341,780 | 5,114 |
|  | 1,346,474 | 1,363,681 | 17,207 |
|  | 1,380,270 | 1,385,275 | 5,005 |
|  | 1,395,664 | 1,399,700 | 4,036 |
|  | 1,406,231 | 1,411,754 | 5,523 |
|  | 1,410,123 | 1,451,733 | 41,610 |
|  | 1,414,374 | 1,421,295 | 6,921 |
|  | 1,435,554 | 1,446,872 | 11,318 |
|  | 1,484,260 | 1,490,476 | 6,216 |
|  | 1,610,814 | 1,618,167 | 7,353 |

| Table S2. Secondary metabolites and antibiotics prediction of Burkholderia contaminans MS14 | | | |
| --- | --- | --- | --- |
|  | Start (bp) | End (bp) | Comment |
| Chr1 | NL30_RS00500 | NL30_RS00585 | Terpene |
|  | NL30_RS01905 | NL30_RS02000 | Terpene |
|  | NL30_RS02655 | NL30_RS02765 | Hse-lactone |
|  | NL30_RS05625 | NL30_RS05715 | Terpene |
|  | NL30_RS06275 | NL30_RS06440 | Phosphonate |
|  | NL30_RS13065 | NL30_RS13170 | Terpene |
|  | NL30_RS14795 | NL30_RS14965 | NRPS: Ornibactin biosynthetic homologous cluster |
| Chr 2 | NL30_RS17965 | NL30_RS18075 | Terpene |
|  | NL30_RS26845 | NL30_RS27030 | Polyhydroxyalkanoate biosynthetic gene homologous cluster |
| Chr 3 | NL30_RS30930 | NL30_RS31005 | Bacteriocin |
|  | NL30_RS32720 | NL30_RS32905 | Pyrrolnitrin biosynthetic homologous gene cluster |
|  | NL30_RS34320 | NL30_RS34520 | Occidiofungin biosynthetic gene cluster |
|  | NL30_RS36125 | NL30_RS36355 | PKS |
|  | NL30_RS37685 | NL30_RS37735 | Bacteriocin |


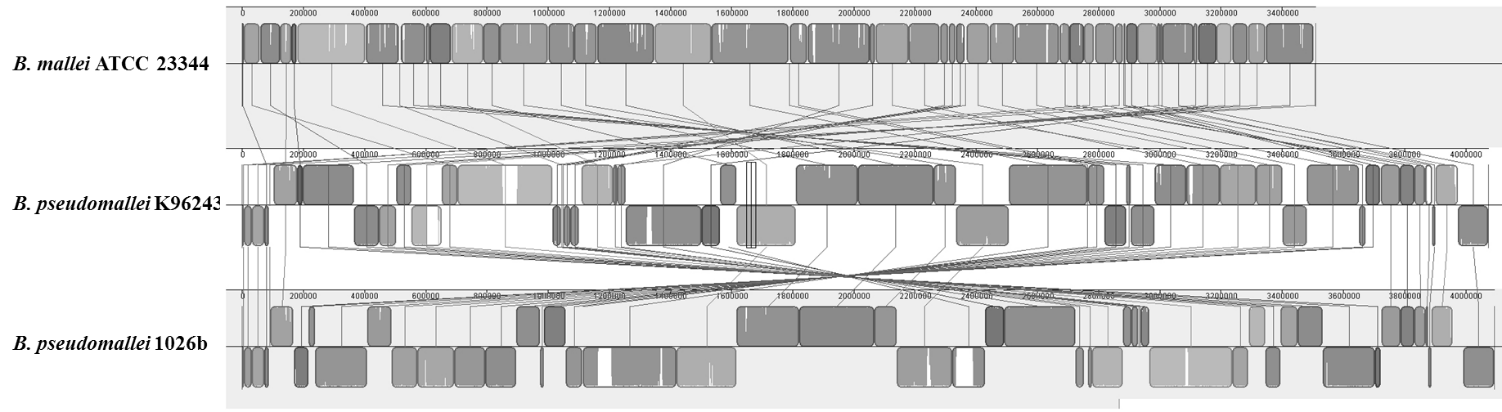


Fig. S1 Synteny of *B. mallei* ATCC 23344, *B. pseudomallei* K96243 and *B. pseudomallei* 1026b chromosome 1. Pairwise alignments of genomes were generated by Mauve, Colored outlined blocks surround regions of the genomic sequence that aligned to other genome. Colored histogram inside blocks indicates the level of sequence similarity. Block lies above the center line the aligned region is in the forward orientation relative to the first genome sequence. Blocks below the center line indicate regions that align in the reverse complement orientation. Regions outside blocks lack detectable homology among the input genomes.
